# Working toward decreasing infant mortality in developing countries through change in the medical curriculum

**DOI:** 10.1186/1447-056X-10-11

**Published:** 2011-08-28

**Authors:** Iffat F Zaman, Ayesha Rauf

**Affiliations:** 1 Department of Pediatrics, Shifa College of Medicine Sector H/8, Islamabad 44000, Pakistan; 2 Department of Medical Education, Shifa College of Medicine Sector H/8, Islamabad 44000, Pakistan

**Keywords:** Curriculum, Infant Mortality, Maternal Mortality

## Abstract

**Background:**

High infant and maternal mortality rates are one of the biggest health issues in Pakistan. Although these rates are given high priority at the national level (Millennium Development Goals 4 and 5, respectively), there has been no significant decrease in them so far. We hypothesize that this lack of success is because the undergraduate curriculum in Pakistan does not match local needs. Currently, the Pakistani medical curriculum deals with issues in maternal and child morbidity and mortality according to Western textbooks. Moreover, these are taught disjointedly through various departments. We undertook curriculum revision to sensitize medical students to maternal and infant mortality issues important in the Pakistani context and educate them about ways to reduce the same through an integrated teaching approach.

**Methods:**

The major determinants of infant mortality in underdeveloped countries were identified through a literature review covering international research produced over the last 10 years and the Pakistan Demographic Health Survey 2006-07. An interdisciplinary maternal and child health module team was created by the Medical Education Department at Shifa College of Medicine. The curriculum was developed based on the role of identified determinants in infant and maternal mortality. It was delivered by an integrated team without any subject boundaries. Students' knowledge, skills, and attitudes were assessed by multiple modalities and the module itself by student feedback using questionnaires and focus group discussions.

**Results:**

Assessment and feedback demonstrated that the students had developed a thorough understanding of the complexity of factors that contribute to infant mortality. Students also demonstrated knowledge and skill in counseling, antenatal care, and care of newborns and infants.

**Conclusions:**

A carefully designed integrated curriculum can help sensitize undergraduate medical students and equip them to identify and address complex issues related to maternal and infant mortality in underdeveloped countries.

## Background

According to current statistics, of the 8.8 million children who die each year, 37% die of neonatal causes. Another 38% die of easily preventable and treatable causes such as pneumonia, diarrhea, malaria, and measles. Globally, more than 24,000 children die every day and every minute one woman dies in childbirth [1].

Pakistan is a developing country with a population of approximately 160 million and is one of the major contributors to the above statistics [2]. In 1990, Pakistan had an infant mortality rate (IMR) of 100 and a child mortality rate (CMR) of 130 per 1000 live births. Maternal mortality ratio (MMR) during that period was 550/100,000. Currently, Pakistan has an IMR of 78 and CMR of 94 [3]. Pakistan's Millennium Development Goals (MDG) aspire to decrease IMR and CMR to 40 and 45 (MDG4) and MMR to 140 (MDG5) by 2015 [4]. It has not even come halfway toward that goal in the past two decades. Clearly, Pakistan will not be able to achieve its targets if the decline in the mortality rates continues to be this slow.

Maternal and child mortality rates are an important determinant of any country's health status. Decreasing maternal and child mortality rates has been given high priority at the national level [4]. Several local and international organizations are working to achieve this end. Many interventions have been suggested and are being tried [5]. Despite these efforts Pakistan has not made any significant improvement in this area and there is a large gap between its goals and the current situation (Table 1.) [6].

**Table 1:** Immunization & Mortality Statistics for Pakistan

Index	1990	2006-07	MGD target
Infant mortality	100	78	40
Child mortality	130	94	45
Fully vaccinated children aged 12-23 months	50%	47%	> 90%

We hypothesize that one of the major factors for this lack of progress is that medical educators are not involved in the attempt to reduce maternal and child mortality rates. Medical educators make little or no effort toward making young doctors and medical students aware of the problem and provide no special training to enable them to handle the situation. Subjects related to maternal and child health are taught disjointedly through different disciplines. Additionally, the textbooks used in our curricula are written in the West where the issues related to infant and maternal mortality are entirely different; thus our medical students and doctors are unaware of the local issues and are not equipped with the knowledge and skills to deal with them.

Medical education in Pakistan consists of 2 years of basic sciences consisting of anatomy, physiology, and biochemistry and three years of clinical training that includes clerkships in departments of medicine, surgery, pediatrics, obstetrics and gynecology, and eye and ENT, and lectures in pathology, pharmacology, community medicine, and forensic medicine. In our traditional medical curriculum topics related to maternal and child health are taught through discrete courses and independent medical disciplines of community medicine in fourth year and obstetrics and gynecology and pediatrics in the final (fifth) year of medical school without any cross-referencing among these subjects vertically or horizontally. The major portion of the curriculum deals with problems of prematurity, congenital malformations, and rare syndromes because these are the common causes of death in the Western world where these textbooks are written [7]. The emphasis as in all subject-based curriculums is on learning the disciplines rather than their application in the real life situations. Pakistan Medical and Dental Council is the regulatory body for undergraduate medical education in Pakistan. Although it now recommends integration of clinical and basic health sciences, to date there have been no reports of the integration of the disciplines of community medicine with obstetrics and gynecology and pediatrics [8].

The goal of this module was to sensitize medical students to the high maternal, infant, and child mortality rates in Pakistan and equip them with knowledge and skills to reduce them. This article describes our efforts to modify the curriculum according to local needs, deliver it in integrated fashion, and assess the students for its effectiveness in achieving its goal.

## Methods

### Organization of the module

Shifa College of Medicine's Department of Medical Education formed a ten-member team consisting of three obstetrician and gynecologists, three pediatricians, two community medicine experts, one pathologist, and one pharmacologist. The team was responsible for defining the goals and objectives of the module, identification of major determinants of maternal, neonatal, and child mortality in Pakistan, development of major themes and subthemes, cases to represent these themes, development of the curriculum, and integration of various departments throughout the process of curriculum development and delivery. The first meeting of the team was held 6 months prior to the delivery of the module. Several formal and informal meetings were held for defining goals and objectives, and for planning curriculum and assessment. The curriculum, timetable, study guide, and proposal for assessment were presented to the faculty in a regular forum for critical analysis. The team also conducted post hoc analysis of the module with the Medical Education Department and presented it at another faculty meeting. The delivery was case-based and occurred mostly in small group sessions. Assessment consisted of multiple choice questions (MCQs), short answer questions (SAQs), and objective structured clinical examinations (OSCE) stations and counseling sessions. We also had a mid-module and post-module focus group with randomly selected students who critically appraised the module, discussed its merits and shortcomings, and gave suggestions for its improvement.

### Curriculum development

The first key decision made by the team was that the objectives of the module, the time allocated for each objective delivery, and its weight in assessment will be directly proportional to its contribution toward maternal and child mortality in underdeveloped countries. The team identified the major causes of maternal, neonatal, and child mortality in underdeveloped countries through a literature review and study of Pakistan Demographic and Health Survey 2006-07. Lack of prenatal care, skilled care during child birth, postnatal care, and breast feeding and immunization are regarded as the most important causes of infant and child morbidity and mortality in underdeveloped countries [1]. By contrast, in the developed world preterm birth is the leading cause of infant mortality, along with congenital malformation, low birth weight, and sudden infant death syndrome [9]. Together, these causes are responsible for 47% of deaths in the neonatal period [10]. Textbooks developed in the West therefore focus on the abovementioned problems [11,12]. Although 99% of all mortality in children aged < 5 years occurs in underdeveloped countries [13] and complications related to delivery alone account for more than one third of neonatal deaths [14], doctors in Pakistan follow the same textbooks that are taught in the West and deal only superficially with major contributors of infant and child mortality in underdeveloped countries. Complications related to pregnancy and childbirth are the most common cause of death in women of childbearing age in Pakistan [6]. Nearly all (99%) maternal mortality occurs in the underdeveloped world; common causes include severe hemorrhage (25%), infections (13%), unsafe abortions (13%), and indirect causes such as anemia and malnutrition (20%). By contrast, < 1% maternal mortality occurs in the developed world [15].

Learning objectives and curricular contents were developed keeping in mind the major contributors of maternal and child mortality in underdeveloped countries. Two major themes, namely, "Safe Motherhood" and "Healthy Child" were identified (see Table 2 and Table 3: Appendix A). Safe Motherhood had several subthemes including problems of expectant mothers in the community, antenatal care, and follow-up of normal pregnancy, postpartum care of the mother, epidemiology of maternal morbidity and mortality, and methods of family planning. The theme Healthy Child contained subthemes including care of normal newborn, neonatal resuscitation, infant nutrition, breastfeeding and weaning, special children in the community, epidemiology of infant morbidity and mortality, expanded program for immunization, and integrated management of childhood illnesses (see Appendix B for sample of detailed curricular content). The curriculum placed special emphasis on awareness of epidemiology and current guidelines for mother and child care. Several clinical scenarios were developed to discuss various problems. Whenever possible, the same scenario was continued with additional information to discuss a new subtheme so as to make the situation more lifelike and elicit greater participation of students. Medical social and ethical problems were built into the cases for identification by the students (see Appendix A).

**Table 2:** LEARNING OBJECTIVES: SAFE MOTHERHOOD

1. Competencies	Module Objectives: Student should be able to	Assessment
Knowledge	1. Describe care of expectant mother (antenatal care)	MCQs, SAQs
	
	2. Describe changes in maternal physiology in normal pregnancy	MCQs
	
	3. Describe pillars of Safe Motherhood	
	
	4. Describe parameters to be evaluated at each visit Describe assessment of fetal growth	MCQs
	
	5. Explain the rationale for different investigations	MCQS
	
	6. Describe methods of family planning, their respective side effects, and contraindications	MCQS
	
	7. Describe strategies to prevent maternal mortality	MCQS, SAQ

Skills	1. Perform maternal counseling	IPE
	
	2. Perform counseling in care of expectant mother	MCQ
	
	3. Perform counseling in family planning	IPE

Scholarship	Perform literature search about common causes of maternal mortality	
	
	Search for ways of decreasing maternal mortality in your community	

Community Health Advocacy	1. Screen for maternal illnesses in community	MCQs
	
	2. Provide nutritional guidance for expectant mother and child	MCQs, SAQs
	
	3. Conduct routine investigation for antenatal care	

Reflective Practitioner	1. Foresee complications	MCQS
	
	2. Devise ways to decrease pregnancy-related complications encountered in the community	

Professionalism	Provide punctuality and empathy; respect patients' rights.	

Communication skills	1. Provide counseling	
	
	2. Explain use of supplements and vaccinations during pregnancy	IPE
	
	3. Explain family planning methods best for different couples	IPE

Collaborative skills	1. Collaborate with community for safe pregnancy and delivery For preventing prenatal conditions predisposing to infant morbidity	

Ethical practice	1. Advocate against illegal abortions	
	
	2. Refrain from prescription of costly supplements	
	
	3. Prevent repeated ultrasound	

Evidence-based lifelong learning	1. Do appropriate literature search for evidence	
	
	2. perform community-based interventions to reduce maternal mortality	

TASKS:

1. Counsel patients regarding normal changes in pregnancy

2. Dietary counseling to patients

3. Husband counseling

4. Antenatal examination (SCIL Lab)

5. Pelvic examination (SCIL Lab)

6. Literature search about common causes of maternal mortality

7. Presentation of literature search

8. Presentation on methods applicable for decreasing maternal mortality in your community

**Table 3:** LEARNING OBJECTIVES: HEALTHY CHILD

Competencies	Module Objectives: Student should be able to	Assessment
Knowledge	Describe routine care of a baby immediately after birth	MCQs, SAQs
	
	Describe neonatal examination and common skeletal and other deformities detectable at birth (TEV, DDH, etc.) Describe APGAR scoring	MCQs
	
	Describe mechanism of breastfeeding and its advantages.	MCQ, IPE
	
	Define malnutrition and describe its classification Discuss methods of assessing child growth and use of growth chart	IPE
	
	Describe Integrated Management of Childhood Illnesses (IMCI) program and its relevance to Pakistan	MCQs
	
	Describe EPI program of Pakistan	MCQ
	
	Define the terms Infant Mortality and Infant Mortality Rate	MCQ
	
	Describe causes of infant mortality and discuss preventive strategies	MCQ

Skill	Counsel about vaccination benefits and side effects	IPE
	
	Counsel about rehabilitation techniques used in care of special children	IPE
	
	Perform routine neonatal care	IPE, in SCIL Lab MCQ
	
	Perform neonatal resuscitation	SCIL Lab, on a dummy neonate
	
	Plot anthropometric measures on growth chart	IPE

Scholarship	Perform research and present papers	
	
	Draw on research for patient care	

Community Health Advocacy	Describe importance of good nutrition, proper vaccination, disease prevention, and problems of malnutrition	MCQ
	
	Describe importance of breastfeeding and appropriate weaning practices	

Reflective medical Practice	Be aware of extreme vulnerability of neonates and take measures to decrease their morbidity and mortality	Viva station, SCIL Lab

Professionalism	Offer punctuality and empathy; respect patient's rights	

Communication skills	Counsel mothers regarding infant nutrition	IPE
	
	Counsel mothers regarding vaccination	IPE
	
	Counsel mothers regarding danger signs; guide mother and midwife for prevention of asphyxia in subsequent babies	MCQ

Collaborative skills	Collaborate in healthy upbringing of child (proper follow-up visits, monitor weight gain and development)	MCQ

Ethical practice	Assist in family planning issues	
	
	Empower the mother	

Evidence-based lifelong learning	Perform literature search on ways to decrease infant mortality	
	
	Incorporate the results to decrease mortality and morbidity of children in the community	

TASKS:

Perform routine neonatal care

Plot anthropometric measurements on growth chart

Perform neonatal resuscitation

Literature search for decreasing infant mortality

Presentation of literature search

Role play on breastfeeding issues

Family planning counseling

Study guides have become a valuable supplement to any module's day-to-day teaching and learning [16]. The study guide we developed consisted of a welcome message explaining to the students the module's importance and what we expected to achieve from it, themes, subthemes, and cases for discussion, organization of each day including time, venues, names of facilitators, references for pre-reading, and e-mail addresses of resource persons (see Appendix B).

### Delivery of the curriculum

The delivery of the curriculum was case-based. Each case cross-linked several subthemes and contained a complete history, physical examination, and investigations. Ten facilitators were assigned for the delivery of this module. Facilitators were relevant team members, registrars from pediatrics and obstetrics and gynecology departments, and instructors from basic health sciences. Faculty in charge of each session discussed the case with the facilitators 2-3 days prior to the sessions and emphasized the important learning points. Additional pre-reading materials were also given to the students when necessary. The class was divided into ten small groups for each session and cases were discussed. A wrap-up session for 10-15 minutes was taken by the session in-charge if s/he so desired (thought necessary) and it was assured that objectives for that session were achieved. Subject boundaries were totally demolished in case development and discussions (see study guide for cases). Large group interactive sessions were conducted for some objectives. Role play was introduced to develop problem solving skills necessary for promotion of breastfeeding. Special problems pertaining to Pakistani social setup such as the influence of parental in-laws and "bad milk" were also discussed. Other strategies used in the delivery of the module were video demonstrations for neonatal examination and hands-on sessions for neonatal resuscitation in the SCIL Lab. A field visit was arranged to a local MCH center. Special emphasis was placed in case delivery on identification of social, ethical, and medical problems by the students. Time allocation for learning strategies was as follows: small group discussion, 60%; large group sessions, 15%; hands-on in SCIL Lab, 10%; role play, 5%; supervised research, 5%; and research presentations, 5%.

## Results

Students were assessed by multiple modalities including written tests with 40 MCQs with five stems and one best answer. These questions regarding infant and maternal mortality and its prevention and the management of morbidity were divided into three categories such as: awareness and basic knowledge, mean score 69.2%; problem identification, mean score 54.6%; and problem solving, mean score 71.4%.

There were also 5 SAQs. The students' mean score of 78% demonstrated adequate absorption of course materials. Another performance test consisted of 6 OSCE and two counseling sessions. The OSCE mean score was 86% showing that they were also knowledgeable and skillful in counseling, antenatal care, and care of newborns and infants. (See Appendix C for sample questions from the exam.) All assessments and feedback demonstrated that the students had developed thorough understanding of the complexity of factors that contribute to maternal and infant mortality. They could identify the situations leading to an increase in mortality and solve the problems presented to them adequately.

### Focus groups, questionnaires, and feedback

Focus groups and written questionnaires were used by the Medical Education Department of Shifa College of Medicine for assessment of each module and its feedback. Evaluation of maternal and child health module focus groups was conducted by a trained facilitator from the Medical Education Department. Ten students were randomly selected for this purpose. No faculty member involved with development or delivery of the module was allowed in this group. The time allocated for the discussion was 1 hour. Focus groups concentrated on the usefulness of the module, evaluation of the facilitators by the students, and strategies to improve the module. A written questionnaire (see Additional File 1: Appendix D) was given to all students at the end of the module with written examination paper. Through the feedback forwarded to us, we learned that students found CMCH 224 a focused and organized module, the facilitators of all the sessions were highly motivating, and their enthusiasm to teach encouraged learning. Students also felt that application of knowledge was emphasized through ensuring involvement of all and community awareness was developed through sessions on immunization, breast feeding, and maternal mortality. Students found ethical and social issues challenging and interesting. Many students were not previously exposed to these issues because of their higher economic and different socio-cultural background. The high point for us in this feedback was the comment by many students that the respect shown to students helped develop their self-esteem and they wanted to demonstrate efficiency.

## Discussion

Improving maternal and child health is a major problem for a large part of the underdeveloped world. Pakistan has been struggling with this problem for several decades now and is falling far behind the goals that it has assigned itself. According to MGD 4, Pakistan aims to reduce its under 5 mortality rate to 45, IMR to 40, and MMR to 140 per 100,000 by 2015 [4]. In Pakistan, many organizations are working to realize MGD 4 and 5. The largest task force involved in this is the system of Lady Health Workers (LHWs). The LHW program is sponsored by the Ministry of Health and consists of approximately 93,000 workers. They are basically 8th graders with a 6-month training course [17]. These health workers work in rural areas and go from door to door for health promotion and family planning. Family planning is an issue fraught with mistrust due to various religious and cultural reasons in Pakistan and this has generated mistrust of lady health visitors and a potentially very good system has not been able to bring any change in maternal and child mortality rates. The Expanded Program for Immunization (EPI) is a program funded by WHO, UNICEF, and many others and run by government of Pakistan [18]. It is a big project launched in 1978 and responsible for free immunization of all children. Free vaccines are supplied to all basic health units. It has made its way into the textbooks of pediatrics followed in most medical colleges but they concentrate on the schedule of EPI and side effects of vaccination only. There is no mention of the importance of vaccination and its impact on reducing infant mortality and morbidity and current burden of vaccine-preventable diseases in Pakistan in local pediatrics textbooks [19]. Disease burden and mortality issues are dealt with by the discipline of community medicine without any horizontal or vertical integration with pediatrics or obstetrics. This may be one reason that the rate of immunization which was 50% in 1990 has now gone down to 47% [20]. Another important project that tries to reduce child mortality and improve maternal health is the Technical Assistance for Capital Building in Midwifery, Information and Logistics (TACMIL) project funded by USAID. Its main functions are influencing the policies, training journalists to address health issues, capital building by 2-3-day workshops, and collecting data but none of these activities involve undergraduate curriculum modification or teaching and learning at an undergraduate level [21]. Pakistan Initiative for Mothers and Newborns (PAIMAN) is a USAID-funded nongovernment organization (NGO) working to improve maternal and child health in Pakistan. According to its official website this NGO involves all relevant stakeholders including government, communities, private sector, and donors. However, it is interesting to note that there is no mention of doctors and medical students or their training in their agenda [22].

Medical educationists have argued for reorientation of institutional systems, structures, and processes to meet local needs [23]. They have rightly argued that dividing medicine into disciplines is an artificial construct. The real world of medical practice is trans-disciplinary in large part [24]. To promote preventive thinking, integration within departments is recommended [25]. A literature search also shows that exposing medical undergraduates to community-based learning early in their curriculum helps sensitize them to community issues and that this cannot be achieved through the traditional curriculum [26].

The Pakistan Medical and Dental Council (PMDC), which is the accrediting body, also recommends integration of preventive and clinical sciences. All important topics that could be helpful in decreasing IMR and MMR are included in the curriculum prescribed by PMDC but they are dealt with disjointedly through the disciplines of pediatrics, obstetrics, and community medicine. They are taught and examined disjointedly without any horizontal or vertical cross linkage [7]. The importance of a maternal and child health module has been recognized and incorporated in learning and teaching in many parts of the world. Many universities in the West are also teaching maternal and child health from the point of view of global health and underprivileged communities. Internet searches led to scores of listing and program details for this module but it is taught not as part of undergraduate medical curriculum but rather in masters in public health programs. We found only one example where, under the guidance of WHO, a maternal and child health module has been incorporated in undergraduate medical education--but nonetheless here again it is taught as a part of community health discipline [27].

## Conclusions

High infant, child, and maternal mortality rates not only are sensitive indicators of any community's health status but also reflect the importance given by society to its most vulnerable members. Maternal and child health issues have been neglected by Pakistani governments and regulatory bodies and also by the medical community. Medical educators have not diverted their energies toward solving this grave problem. Medical colleges in Pakistan still spend a large portion of their meager resources in teaching medical students about problems described in Western textbooks. These problems are not the major contributors of maternal or child mortality in underdeveloped countries. For the undergraduate curriculum to be meaningful in addressing these issues in underdeveloped countries, it needs to be modified according to local needs and delivered in integrated fashion. In the development of this module for our undergraduate students, the basic ideas were modification of the curriculum to serve local needs, integration of various clinical science departments with no subject boundaries, and promotion of preventive thinking. We received positive feedback from students regarding this module through focus groups. Assessment of the students done by various modalities showed a deep understanding of the complex problems contributing to high maternal and child mortality in Pakistan. They also demonstrated adequate skills to prevent and manage common problems encountered in maternal and child care. We hope that if medical educators start modifying their curricula to address local needs, it will go a long way toward solving burning health issues in their communities.

## List of Abbreviations

CMR: child mortality rate; IMR: infant mortality rate; LHW: Lady Health Workers; MDG: Millennium Development Goals; MMR: maternal mortality ratio; TACMIL: Technical Assistance for Capital Building in Midwifery and Logistics; USAID: United States Agency for International Development

## Competing interests

The authors declare that they have no competing interests.

## Authors' contributions

IZ was the team leader for development of the module. She also conducted the major portion of the research on causes of infant and maternal mortality in underdeveloped countries. AR conducted focus group discussion and analysis of feedback questionnaire. Both authors approved the manuscript.

## Authors information

IZ is associate professor of pediatrics at Shifa College of Medicine, closely associated with the development of the integrated curriculum, and has run the MCH module three times. Her special interest within pediatrics is child nutrition.

AR is a coordinator in the Department of Medical Education. Her work involves assessment of different modules for their effectiveness and impact on students.

## APPENDIX A

Sample from study guide

SAMPLE CASES

**Subtheme: care of a pregnant woman: **A 26-year-old woman visits your clinic with complaints of nausea and vomiting for the past 1 week. Her last menstrual period was 8 weeks previously. On missing her periods she did her home urine pregnancy test, which came out as positive. Now for the past 1 week she has early morning nausea and vomiting. She also feels dizzy and weak and is unable to perform her chores normally.

**Subtheme: postpartum care of mother: **Sajida is brought into the hospital emergency room 6 days after her delivery with history of high-grade fever for 3 days, heavy vaginal bleeding for 4 days, and burning micturation for 5 days. She reports that after going home she has not been feeling well. She stopped taking all medications advised to her at hospital because her mother-in-law told her so. On examination, she is very pale, dehydrated, and weak. Her BP is 90/40 mmHg, pulse 126/min, temp 102°F. Her chest is clear and there is tenderness in her lower abdomen. Speculum examination reveals foul smelling discharge. On bimanual examination uterus is 18 weeks size and tender. Her left calf is swollen, hot, and painful.

**Subtheme: maternal mortality: **Farieda is a 40-year-old woman married to Hassan for the last 25 years. She and Hassan lived in the Northern Areas beyond the Hunza valley in an area called Sost. Her family moved to Rawalpindi 3 years ago because they were unable to sustain their living on the small piece of land that Hassan owned. Farida's mother-in-law and an unmarried sister-in-law live with the couple along with her 8 children.

All of Farieda's children until the seventh were born at home by a traditional birth attendant in Sost. She wanted to stop getting pregnant after she had her fourth child. She was unable to visit the family planning center because the mother-in-law forbade her because she wanted Hassan who was an only son to have more sons.

Farieda and her family moved into a slum area of Rawalpindi. Her husband obtained work as a security guard and does double duty to sustain his family by working 12-15 hours daily. She works as a maid in 6 households. Her elder daughter takes care of the home and her younger siblings. She had her last child at Shifa Foundation Clinic and the child is currently 10 months old. She breastfed for a few months but was unable to continue because her milk was not sufficient especially after working for a long day. This time she presented to Shifa Foundation Clinic again with amenorrhea for 8 weeks and was found pregnant. She requested the obstetrician for an abortion but the doctor did not oblige but was sympathetic to Farieda and counseled her and offered her support to continue the pregnancy. On examination she was pale and weighed 40 kg (her height was 5 feet 2 inches). The doctor advised her to take iron tablets after checking her hemoglobin, which was 6 mg/dl. She was unable to tolerate the tablets because of GI upsets so she was not compliant with the drugs. She received tetanus injections in her antenatal visits. She developed high blood pressure during one of her visits and was advised bed rest and antihypertensive medications. During her 8th month and still with high blood pressure, the doctor advised her to take bed rest but she gave a sarcastic laugh and said "Doctor Jee, if I go on bed rest who will earn for the family? How will so many mouths be fed? How can I take rest?"

**Subtheme: infant mortality: **Farieda brings her 2-month-old baby girl to hospital with 4-day history of reluctance to feed and 3-day history of shortness of breath. She was giving the baby water and honey on the advice of her elders at home. She brought the baby to the hospital when she realized that the baby was becoming worse despite this treatment. Birth weight was not known because she was born at home with no skilled help. Farieda says that at birth the child appeared small but had cried right after birth. Baby on examination was weak and pale. Her weight was 2.7 kg. Her respiratory rate was 80 breaths/minute, heart rate 162 beats per minute, and temperature 96°F. Her oxygen saturation was 73%. She was worked-up for sepsis and pneumonia and was started on broad-spectrum antibiotics. She died 16 hours after admission.

## APPENDIX B

(Sample from detailed curricular content)

CURRICULAR CONTENT

1. Maternal Health in Community

**KNOWLEDGE: **Importance of mother's profile on the health of mother and fetus, effect of maternal age, consanguinity, maternal profession, radiation exposure, chemicals, toxins, smoking, alcohol, drugs, medications, nurses, doctors (hepatitis B and C vaccination) gravidity, parity, abortions age of previous child/place of birth/mode of delivery/reason for that mode/duration of pregnancy/complications of pregnancy, labor, and puerperium/sex of baby/weight of baby

**SKILL: **History of present complaints in chronological order, past obstetrical and gynecological history, family history, history of current social problems and other health issues not volunteered by mother (tuberculosis, diabetes mellitus, hypertension, heart disease, renal disease), addictions: drugs, smoking, alcohol, substance abuse

**ATTITUDE: **Sensitization and development of a sympathetic approach towards problems common among women in Pakistan

**TASK: **Take one history from foundation clinic and identify at least three problems. Submit the history on following Monday

THEME: Antenatal Care

Present state of health/immunization, appropriate maternal weight gain, fetal movements, fetal growth (fundal height measurement, fetal heart auscultation, fetal growth and wellbeing, any maternal or fetal complaints (vaginal discharge, burning in urine, dyspnea, fever, decreased fetal movements), mother's nutrition and supplements: maternal malnutrition and obesity, anemia, role of folic acid, iron, and calcium, balanced diet, breastfeeding sensitization: role of breastfeeding in growth development and disease prevention in the child

**Complications and referrals: **Headaches, raised blood pressure, fundal height (more or less than dates) effect of maternal diseases on fetal development (diabetes, hypertension, renal disease, hyperthyroidism, hypothyroidism, TORCH-S, HIV, hepatitis infections)

**Antenatal examination**: Height, weight, complete general physical examination (vital signs, palor, jaundice, cyanosis, thyroid, breasts, edema) and systemic examination (special emphasis on abdominal examination and pelvic examination--speculum and bimanual), any complications

**SKILL: **Abdominal examination, SCIL Lab; pelvic examination, SCIL Lab

**Attitude**: Sympathetic approach towards mothers

APPENDIX C (Sample questions from the exam)

Sample questions from the module

A woman presents with a history of termination of previous pregnancy because of fetal anencephaly. Karyotyping was done and was normal. She is planning to get pregnant again. On examination she is pale looking but otherwise normal. Which of the following will most likely prevent similar outcome in future pregnancies?

a. Ferrous sulfate 60 mg daily

b. Vitamin B complex

c. Calcium 1 g daily

d. Folic Acid 5 mg daily*

e. Vitamin E 100 mg daily (**Problem solving)**

A baby boy was born at 37 weeks of gestation with prolonged rupture of membranes for > 30 hours. The mother was afebrile and not treated with antibiotics. Postnatally, the woman complains of severe lower abdominal pain and offensive blood discharge. Most probable diagnosis is:

A. Urinary tract infection

B. Pelvic inflammatory disease

C. Cervicitis

D. Vaginitis

E. Appendicitis **(Problem identification)**

The baby assessed by on call doctors was well. The most appropriate step in the management of this baby would be:

A. Discharge the baby now and follow-up after 1 week

B. Observe the baby in hospital for 48 hours

C. Wait for the result of vaginal swab then treat the baby accordingly

D. Do the blood culture of the baby and start antibiotics if culture is positive

**E. Do the blood culture and treat the baby with I/V antibiotics **(**Problem solving)**

## Supplementary Material

Additional file 1 **Appendix D- Feedback Questionnaire**. Click here for file

## References

[B1] Global Action for Children http://issuu.com/scarkonen/docs/gac_2009_annual_report_web (accessed August 8, 2010.)

[B2] Index Mundi http://www.indexmundi.com/map.aspx?v=Infant+mortality+rate%28deaths%2f1%2c000+live+births%29&co=as

[B3] Lalji Najma Thaver Ali Minhal Kamal Ameera Maternal Neonate and Child Health (MNCH) research in Pakistan: Trend and transition J Pak Med Assoc 2010 60 5 401 403 http://20527620

[B4] UNICEF Millenium Developement Goals http://www.ncbi.nlm.nih.gov/pubmed/20088466

[B5] Bhutta ZA Gupta I DeSilva H Manandhar D Awasthi S Hussain MS Maternal and child health: is South Asia ready for change? BMJ 2004 328 816 819 10.1136/bmj.328.7443.816 15070640 PMC383381

[B6] 2006-07 Pakistan Demographic and Health Survey Final Report http://www.measuredhs.com/pubs/pub_details.cfm?ID=783

[B7] Mathews TJ MacDorman MF National Vital Statistics Report http://www.cdc. gov/Nchs/data/nvsr/nvsr57/nvsr57_02.pdf

[B8] Pakistan Medical and Dental Council http://www.pmdc.org.pk/LinkClick.aspx?fileticket=EKfBIOSDTkE%3d&tabid=102&mid=556

[B9] Simhan HN Preterm birth is the leading cause of neonatal mortality and is responsible for roughly one-half of long-term neurologic sequelae Am J Obstet Gynecol 2010 202 407 408 10.1016/j.ajog.2010.03.027 20452479

[B10] Kung HC Hoyert DL Xu JQ Murphey SL E-Stats Deaths: Preliminary Data for 2005 Health E-Stats http://www.cdc.gov/nchs/products/pubs/pubd/hestats/prelimdeaths05/prelimdeaths05.htm

[B11] Behrman RE Kliegman RM Jenson HB (Eds) Nelson Textbook of Pediatrics 2000 16 Philadelphia: Harcourt Brace

[B12] Oski FA DeAngelis CD Feigin RD Warshaw JB Principles and Practice of Pediatrics 1990 Philadelphia: JB Lippincott

[B13] The State of the World's Children--Child Survival http://www.unicef.org/sowc08/report/report.php

[B14] Nour MN An introduction to maternal mortality Rev Ob Gyn 2008 1 77 81 PMC2505173 18769668

[B15] US Coalition for Child Survival http://www.child-survival.org/advocacy/gcsa.php

[B16] Gareth J Hoslgrove JH Lanphear IL Study guide: an essential student learning tool in an integrated curriculum Med Teach 1998 20 99 103 10.1080/01421599881174

[B17] National Programme for Family Planning & Primary Health Care http://www.phc.gov.pk/site/lady-health-workers.html

[B18] Expanded Program on Immunization (EPI) http://www.epipak.org (accessed August 19, 2011)

[B19] Khan PA Kundi Z Basis of Pediatrics 2005 7 Lahore: Zahid Bashir

[B20] Bhutta Z Cross A Raza F Zahir Z Measure Demographic and Health Surveys http://www.measuredhs.com/pubs/pdf/FR200/FR200.pdf

[B21] TACMIL Health Project http://www.usaid.gov/pk/sectors/health/tacmil.html (accessed August 19, 2011

[B22] Pakistan Initiative for Mothers and Newborns (PAIMAN) http://pdf.usaid.gov/pdf_docs/PDACH527.pdf

[B23] Jones R Pitama S Huria T Poople P McKimm J Medical education to improve Maori health NZ Med J 2010 123 113 122 20648107

[B24] Smith SR Towards an integrated curriculum Med Health RI 2005 88 258 261 16273968

[B25] Cheng TL Greenberg L Helen LH Keller D Teaching prevention in pediatrics Acad Med 2000 75 7 Suppl 566 571 10.1097/00001888-200007001-00010 10926043

[B26] Buckner AV Ndjakani YD Banks B Blumenthal DS Using service-learning to teach community health: the Morehouse School of Medicine Community Health Course Acad Med 2010 85 1645 1651 10.1097/ACM.0b013e3181f08348 20881688 PMC3976958

[B27] Searo EG Review of preventive and social medicine, community medicine and community health curriculum for undergraduate medical education social medicine 2009 New Dehli: WHO Regional Office for South East Asia

