# Supplementary material for: Working toward decreasing infant mortality in developing countries through change in the medical curriculum
Source: Asia Pac Fam Med. 2011 Aug 28;10(1):11. doi: 10.1186/1447-056X-10-11 (PMC3180395; doi:10.1186/1447-056X-10-11)
Supplement: Additional file 1 — Appendix D- Feedback Questionnaire. [file 1447-056X-10-11-S1.DOC]

**APPENDIX D**

**Feedback questionnaire used**

**Shifa College of Medicine**

**Module Feedback Form**

**Class: __________ Module Name: ____________________________ Date: __________**

1. What was the impact of this module on your learning? (stimulating, probing, reflective thinking, clinical reasoning, raised more questions than providing answers)
2. How do you relate the learning during this module with solving patient-related problems? (learned to apply knowledge in solving patient-related problems)
3. Which aspects of ethics and professionalism were specifically highlighted in this module? (ethical dilemmas addressed that may arise due to new knowledge or situations, professional aspects in dealing and communicating with patients)
4. What skills learned in the module will be helpful in dealing with peers and patients? (team work, collaborative projects, leadership, empathy, virtues highlighted to deal with patients and peers)
5. Did the module help your thinking in a different perspective (linking with other modules, learning more than knowing) or help in self-assessment (addressed ways to self-assess in terms of knowledge, skills, and attitudes)?
6. How did the module delivery help you realize the use of updated research evidence in effective health care delivery? (evidence presented, questions raised that prompted you to look at updated research)
7. Rate faculty members involved in this module based on their inspiring behaviors, learning experiences, mentoring, and/or motivation)

Name of faculty member (The least) 1 2 3 4 5 (The most)
